# Post-Transcriptional Expression Control in Platelet Biogenesis and Function

**DOI:** 10.3390/ijms21207614

**Published:** 2020-10-15

**Authors:** Carolin T. Neu, Tony Gutschner, Monika Haemmerle

**Affiliations:** 1 Institute of Pathology, Section for Experimental Pathology, Medical Faculty, Martin-Luther University Halle-Wittenberg, 06120 Halle/Saale, Germany; carolin.neu@uk-halle.de; 2 Junior Research Group ‘RNA Biology and Pathogenesis’, Medical Faculty, Martin-Luther University Halle-Wittenberg, 06120 Halle/Saale, Germany; tony.gutschner@uk-halle.de

**Keywords:** RBP, miRNA, lncRNA, circRNA, platelets, thrombosis, posttranscriptional regulation

## Abstract

Platelets are highly abundant cell fragments of the peripheral blood that originate from megakaryocytes. Beside their well-known role in wound healing and hemostasis, they are emerging mediators of the immune response and implicated in a variety of pathophysiological conditions including cancer. Despite their anucleate nature, they harbor a diverse set of RNAs, which are subject to an active sorting mechanism from megakaryocytes into proplatelets and affect platelet biogenesis and function. However, sorting mechanisms are poorly understood, but RNA-binding proteins (RBPs) have been suggested to play a crucial role. Moreover, RBPs may regulate RNA translation and decay following platelet activation. In concert with other regulators, including microRNAs, long non-coding and circular RNAs, RBPs control multiple steps of the platelet life cycle. In this review, we will highlight the different RNA species within platelets and their impact on megakaryopoiesis, platelet biogenesis and platelet function. Additionally, we will focus on the currently known concepts of post-transcriptional control mechanisms important for RNA fate within platelets with a special emphasis on RBPs.

## 1. Introduction

Blood platelets—the major players in hemostasis—are small anucleate cell fragments with a characteristic discoid shape and a diameter of 1 to 3 µm that originate from megakaryocytes (MKs). Platelets are the second most abundant “cell” type in the peripheral blood and constitutive renewal ensures a normal platelet count in the blood, despite their relatively short life span of 7–10 days [1,2]. Platelets are also interesting from a clinical point of view. Platelet counts, which range from 150,000 to 350,000/µL of whole blood in healthy subjects, can drastically increase and decrease in pathophysiological conditions, resulting in thrombocytosis or thrombocytopenia, respectively [3]. Thus, it is not surprising that a wide range of human pathologies are associated with abnormal platelet counts and/or functions like inflammatory diseases [4], rheumatoid arthritis [5], diabetes [6], pulmonary hypertension [7], Alzheimer’s disease [8], cardiovascular disease [9], and cancer [10,11].

The unique life cycle of platelets starts with proplatelet biogenesis from MKs in response to certain stimuli. MKs develop from hematopoietic stem cells (HSCs) present in the bone marrow, yolk sac, fetal liver, and spleen [12]. As a prerequisite, MKs initially have to grow and multiply their genetic information via endomitosis. These processes are highly dependent on thrombopoietin (*TPO*), a cytokine that promotes the growth and development of MKs from their precursors and that associates with its MK-specific receptor, i.e., cluster of differentiation 110 (*CD110*), encoded by the cellular myeloproliferative leukemia virus (c-MPL) oncogene [13,14,15]. Both *c-Mpl*^−/−^ and *Tpo*^−/−^ mice are thrombocytopenic and have reduced numbers of MKs in the bone marrow [16,17,18]. However, although platelet and MK numbers were significantly reduced, the remaining MKs and platelets were structurally normal and platelets were functional as they were still able to form clots and responded to agonists [19]. This suggests that other regulatory factors exist [20]. Furthermore, endomitosis, a process during which MKs accumulate DNA content ranging from 2N to 128N in their big polylobulated nucleus, also contributes to platelet formation and the degree of MK ploidy was shown to influence platelet quantity and quality [21,22]. Mechanistically, endomitosis is a process by which MKs become polyploid through repeated rounds of DNA replication without cell division, which requires both coordination and restriction of cell cycle components [23,24,25]. Importantly, endomitosis is triggered by *TPO* and is necessary for MKs to proceed with their final maturation and subsequent proplatelet formation [22]. Another purpose of endomitosis is to produce large amounts of proteins and lipids necessary for the MK demarcation membrane system (DMS) that serves as a membrane reservoir for proplatelet formation. The origin of the DMS likely involves invaginations of the MK plasma membrane, de novo membrane synthesis, as well as contributions from the Golgi-derived membranes and close endoplasmic reticulum (ER) contacts [26]. In the final step of platelet biogenesis, terminally differentiated MKs remodel their cytoplasm to form long protrusions, so-called proplatelets, which extend into bone marrow sinusoids and are the progenitors of mature platelets [27]. However, terminal platelet formation might not end in the bone marrow sinusoids, but likely continues in the circulation and was shown to be substantial in the lung [28,29]. Of note, in an attempt to delineate the final steps of proplatelet maturation and platelet release, a large (2–10 µm) intermediate discoid stage in platelet production, the preplatelet, was identified [30]. Preplatelets are anucleate discs that have a thick cortical microtubule coil and are able to convert back into barbell-shaped proplatelets. Final platelet release is achieved through continued bidirectional polymerization of microtubules at each end of the proplatelet followed by a fission event. This multi-step biogenesis yields approximately 100 billion platelets each day in an adult human, but these rates can increase by a factor of 10 or more when the demand increases [31]. Interestingly, in recent years ex vivo protocols have been developed to generate functional platelets from megakaryocytes, which are differentiated from progenitor/stem cells. These models are generally based on bioreactors with biocompatible porous barriers that support MK function and platelet release is supported by media flow obtained by electronic pumps. These tissue models are especially important because platelet transfusions are common, but supply does not always match demand [32,33]. However, if cultured platelets are to become a considerable clinical alternative, they must match the quality and functionality of donor-derived platelets. Therefore, a better understanding of the mechanisms responsible for MK maturation, platelet generation and release is necessary [34].

Mature platelets were first discovered by Bizzozero in the late 19th century and have since been ascribed a primarily hemostatic role. While circulating in an inactive state in the blood stream, platelets become activated upon exposure to subendothelial collagen to support thrombus formation, highlighting their outstanding role as first responders in wound healing and hemostasis [35]. Normally, intact endothelium expresses and releases factors that prevent platelets from activation and aggregation and block fibrin clot formation. After endothelial injury, subendothelial collagen gets exposed to the blood and initiates activation and adhesion of platelets via von Willebrand factor. Expression of tissue factor (TF) finally activates a sequence of events to establish a stable clot that seals the injury site and prevents excessive blood loss [36]. In the last centuries, it has become evident that platelets are not only implicated in hemostasis and thrombosis, but also act as key players in infectious and sterile inflammatory responses as well as cancer [11,37]. Many of the non-hemostatic functions of platelets result from the storage and release of bioactive molecules. These molecules, be they platelet proteins, lipids, growth factors, or cytokines, are mainly found in alpha (α) and dense (δ) granules [38]. The biogenesis of these granules begins in the MK, but maturation continues in circulating platelets [39,40]. Initially, it was hypothesized that granule packaging and release is a random process, however, recent studies suggested that platelets contain heterogeneous populations of granules that differentially release their content upon different physiological stimuli [41,42].

Besides their protein content, platelets contain different nucleic acids including small and long non-coding RNAs and protein-coding messenger RNAs (mRNAs). First thought to be incapable of regulated gene expression, researchers started to shed light on the translational machinery and its regulation in platelets in the absence of a nucleus. Subsequent studies revealed that platelets possess a functional spliceosome, allowing for rapid adaptations to changing environmental cues and a change of phenotype [43]. In recent years, in-depth transcriptional analyses of platelets have been performed suggesting that as many as 3000–6000 mRNAs are contained in platelets [44,45,46]. However, the most intriguing question is: How and where do platelets get their content from? While the precise mechanisms are still largely unknown, it is becoming evident that the inheritance of proteins and mRNAs from the megakaryocytes is not a random but highly regulated process.

This review will highlight the different classes of RNA species enriched in platelets, will focus on the role of non-coding RNAs in platelet biogenesis and function and will feature the role of post-transcriptional control mechanisms with a special emphasis on the role of RNA-binding proteins.

## 2. Composition and Sources of RNA Species in Platelets

**Figure 1:**
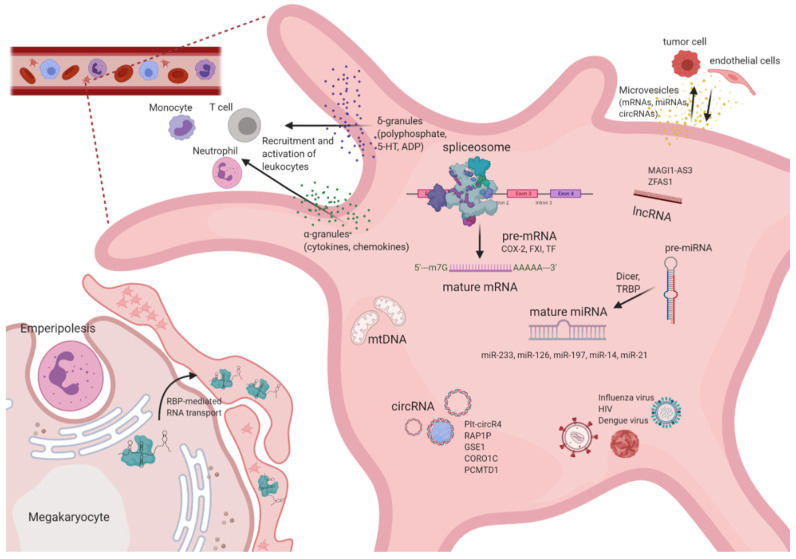
Nucleic acids landscape of platelets. Platelets harbor a diverse set of RNA species, most of which are inherited from megakaryocytes. Additionally, communications with surrounding cells might also influence RNA expression landscape. Transport of RNA from megakaryocytes to platelets is not well understood, however, is suggested to be—at least in part—mediated by RNA-binding proteins (RBPs). Pre-mRNA and pre-miRNA are processed by a functional spliceosome and by the presence of the cytoplasmic miRNA processing into translate mRNA and mature miRNA, respectively, which can be signal-dependent. Platelets may also host infectious virus particles and mediate dissemination of viruses in infected individuals. By secreting RNA and protein contents by platelet granules or microvesicles, platelets may regulate signaling pathways in various interacting cells, including tumor or endothelial cells.

A dynamic transcriptome is a prerequisite for platelet function, enabling rapid responses to external stimuli. However, RNA abundance in platelets is ~1000 times less than in leukocytes, leading to challenges when it comes to transcriptome analysis of platelets, as high purity samples are crucial. Despite these challenges, independent studies have found a diverse set of nucleic acids within platelets such as different classes of RNAs [43,47,48,49,50] as well as mitochondrial DNA (mtDNA) [45]. Interestingly, it has been observed that the content of total RNA decreases over time and young, reticulated platelets show 20–40 times higher RNA levels compared to older ones, which indicates either decay and/or release of RNA during the lifespan of platelets [51]. The bulk of platelet RNA is derived from megakaryocytes, probably involving active transport mechanisms for sorting into platelets. In addition, other cellular sources might exist as well given the intimate interaction of platelets with other cell-types in the circulation or within tissue microenvironments. Figure 1 summarizes the plethora of RNA molecules in platelets including their source and additionally highlights the importance of intercellular communication via granule release and microvesicle shedding.

### 2.1. RNA Molecules in Platelets

#### 2.1.1. Messenger RNAs (mRNAs)

Among the different types of RNA, mRNAs are the best investigated class in platelets. They present typical features of eukaryotic mRNAs like a 7-methylguanosine (m^7^G) 5′ cap followed by a 5′-untranslated region (UTR) as well as a 3′-UTR and poly(A)-tail at their 3′-end. The m^7^G cap and poly(A) tail promote mRNA translation and stability, while the UTRs expose sequences for RNA-binding proteins (RBPs) and regulatory sites for microRNA (miRNA)-mediated translational and degradation control [45,52,53,54]. Getting a complete picture of the (dynamic) nature and composition of the platelets transcriptome is of great interest and next generation sequencing (NGS) has proven to be one of the preferred techniques for such studies [55,56]. Bulk platelet analyses demonstrated that platelets harbor a diverse set of mRNAs and showed an enrichment of genes functionally linked to coagulation, platelet degranulation, cytoskeletal dynamics, receptor binding, secretion, and G-protein signaling, all being a prerequisite for a plethora of signaling pathways. Along these lines, it is worth mentioning that platelets were shown to contain also pre-mRNAs as well as small nuclear RNAs (snRNAs), which are functionally linked to gene expression and involved in pre-mRNA splicing. In fact, Denis et al. could show that platelets contain a complete set of snRNAs as well as other essential splicing factors and are capable of signal-dependent splicing. Here, in response to integrin engagement and surface receptor activation, platelets precisely excised introns from interleukin *(IL)-1β* pre-mRNA, thereby generating a mature mRNA transcript that was translated into protein [43]. Until then, it has been thought that splicing events only occur within nuclear boundaries, which has been challenged by these findings. However, since the initial discovery of a functional spliceosome in platelets, several other studies confirmed the existence and functional importance of splicing for platelet activation. For example, splicing upon platelet stimulation has also been shown for platelet factor XI (*FXI*) pre-mRNA and significantly higher amounts of pre-mRNA were found in resting compared to thrombin-activated platelets [57]. Moreover, activation of platelets by fibrinogen and thrombin induced splicing of tissue factor (*TF*) pre-mRNA leading to an increased TF protein expression, procoagulant activity, and accelerated formation of clots. Mechanistically, it was revealed that TF pre-mRNA splicing was dependent on Cdc2-like kinase (Clk)1-mediated Splicing Factor 2 (SF2) phosphorylation [58]. Furthermore, lipopolysaccharide (LPS) was shown to induce splicing, translation, and secretion of mature *IL-1β* through TLR4 [59]. Similarly, LPS stimulation of platelets initiated splicing of cyclooxygenase-2 (*COX-2*) pre-mRNA and ultimately production of the corresponding protein. However, this effect was extremely variable and it appeared that LPS stimulated platelets either produced very little or non-active COX-2 protein [60]. Of note, signal-dependent splicing, but also mRNA degradation and translation might explain controversial findings regarding correlation between mRNA and protein expression in platelets with some studies suggesting a weak correlation of the platelet transcriptome and proteome [61], while others report a rather strong correlation, opening up the question on regulatory mechanisms of mRNA metabolism in platelets under physiological and pathological conditions [62].

Another intriguing aspect of the platelet transcriptome is the fact that the RNA content of platelets is significantly influenced by interactions with surrounding cells including cancer cells. For example, Best et al. recently discovered that tumor cells are able to educate platelets and induce an RNA expression signature within platelets that could be used for non-invasive cancer diagnostics [63,64,65,66]. Moreover, a standardized protocol was established to enable isolation and analysis of spliced platelet mRNA, which allowed detection of cancer with high accuracy [63]. This method has the potential to become clinically relevant, as platelet isolation is a rather simple standard procedure, already established in hematology laboratories. Importantly, small amounts of only 100–500 pg of total platelet RNA were sufficient for diagnostics and allowed separation of cancer patients from healthy individuals with 96% accuracy using mRNA sequencing [64]. Additionally, platelet RNA signatures helped to distinguish patients with Kirsten Rat Sarcoma Viral Oncogene Homolog (*KRAS*), Epidermal Growth Factor Receptor (*EGFR*) and Phosphatidylinositol-4,5-Bisphosphate 3-Kinase Catalytic Subunit Alpha (*PIK3CA*) mutant and wild-type tumors, as well as patients with Human Epidermal Growth Factor Receptor 2 (*HER2*)-amplified versus HER2-negative breast cancer and Mesenchymal Epithelial Transition tyrosine kinase (*MET*) overexpressing versus *MET* non-overexpressing lung cancers [64]. Although these studies seem promising, there are still some obstacles to be overcome such as the distinction between primary and metastatic tumors and the evaluation of cancer progression. One should also be aware of the fact that non-cancerous systemic factors like inflammation and cardiovascular events could influence platelet RNA profiles as well [67]. However, using platelet-based mRNA expression signatures in various diseases including cancer could open up new diagnostic avenues.

Taken together, RNA expression profiling of platelets increased our understanding of the quantity and quality of their RNA content. However, future studies are needed to shed new light onto the functional roles of these mRNAs and their encoded proteins or whether these mRNAs are simply the result of an inflammatory and/or pro-oncogenic environment.

#### 2.1.2. MicroRNAs (miRNAs)

Another class of RNAs present in platelets is microRNAs (miRNAs). These 21–24 nucleotide (nt) spanning microRNAs (miRNAs) are known key regulators of eukaryotic gene expression [68]. Alteration of miRNA expression signatures has been observed in cancer and might predict prognosis and aid in diagnosis [69]. Additionally, miRNAs are important regulators of MK differentiation from hematopoietic progenitor cells [70]. Microarray and RNA-Sequencing (RNA-Seq) analysis identified up to 532 different miRNAs in human platelets [71]. Interestingly, post-transcriptional modifications like adenylation and uridylation that are known for their regulatory impact on miRNA stability and decay, also seem to play a role in miRNA biology in platelets [72]. The relevance of miRNAs in platelets has been recognized more and more in recent years and was particularly fueled by the discovery of a functional miRNA processing machinery in platelets [49]. In general, miRNA biogenesis starts with the transcription of primary miRNAs (pri-miRNAs), which are subsequently processed into precursor miRNAs (pre-miRNAs). A prerequisite for these initial steps is the micro-processor complex, consisting of nuclear RNase III Drosha and the DiGeorge syndrome critical region 8 (DGCR8), which is localized in the nucleus of cells [73]. Following nuclear export, the cytoplasmic RNase III enzyme Dicer and Trans-Activation Responsive RNA-Binding Protein 2 (TARBP2) cooperatively cleave the stem of pre-miRNA substrates yielding mature miRNAs [74,75,76,77]. Landry et al. were the first to describe the cytoplasmic pre-miRNA processing machinery in human platelets, including Dicer and Argonaute 2 (Ago2) proteins [49]. In contrast, the nuclear microprocessor components Drosha and DGCR8 could not be detected, consistent with the anucleate nature of platelets. Using RNase activity assays, it was demonstrated that platelet Dicer was functional and able to convert pre-let-7a-3 into mature miRNA. Additionally, platelet miRNAs were able to mediate RNA silencing as exemplified by the regulation of the purinergic receptor *P2Y12*, which is involved in platelet aggregation, through an Ago2/miR-223 effector complex contained in platelets [49]. Moreover, functional Ago2/miR-223 effector complexes were packaged into microparticles and released by platelets upon thrombin stimulation and subsequently internalized by endothelial cells, where they regulated F-Box and WD Repeat Domain Containing 7 (*FBXW7*) and Ephrin A1 (*EFNA1*) mRNA and protein levels [78]. Additionally, platelet-secreted miR-223 was shown to promote endothelial cell apoptosis induced by advanced glycation end products via targeting the Insulin-like Growth Factor 1 Receptor (*IGF1R*) [79]. Moreover, RNA-Seq analysis of platelet miRNAs in patients with myocardial infarction revealed nine differentially expressed platelet miRNAs compared to healthy controls, which were released upon platelet aggregation and taken up by endothelial cells via a vesicle-dependent mechanism [80]. Importantly, microvesicle-mediated miRNA transfer to endothelial cells altered endothelial cell behavior and did not only influence endothelial cell survival but also inhibited tube formation as it has been shown for platelet-derived miRNA let-7a, which targeted Thrombospondin-1 (*THBS-1*) 3′-UTR and inhibited THBS-1 release from endothelial cells [81]. Next to endothelial cells, cancer cells also are recipients of platelet miRNAs. For example, miR-223 expression was found to be significantly increased in platelets and platelet-derived microvesicles from non-small cell lung cancer (NSCLC) patients as well as from mice intravenously injected with Lewis lung carcinoma cells. Of note, platelet microvesicles effectively delivered miR-223 to lung cancer cells and promoted cancer cell invasion by reducing Erythrocyte Membrane Protein Band 4.1-like 3 (*EPB41L3*) levels [82]. These examples highlight the broad range of cell-platelet communication via miRNAs, not only in the context of cancer. In fact, the majority of circulating miRNAs derived from platelets and altered signatures of circulating miRNAs were identified in patients with type 2 diabetes mellitus and with future myocardial infarction [83,84,85]. Antiplatelet therapy using aspirin and prasugrel significantly decreased the level of platelet miRNAs suggesting that plasma miRNA expression could be used as surrogate markers of platelet activation and efficacy of antiplatelet therapy [83]. Interestingly, the potential of miRNAs as biomarkers of platelet activity in antiplatelet therapy monitoring has been recently reviewed elsewhere [86].

In summary, platelets inherit a functional miRNA pathway from their mother cells which enables a complex crosstalk and post-transcriptional gene expression control based on the regulatory nature of miRNAs [49]. Thus, platelet-secreted miRNAs are important modulators of signaling pathways in target cells and could serve as disease biomarkers, or as activation and therapy response markers.

#### 2.1.3. Long Non-Coding RNAs (lncRNAs)

Long non-coding RNAs (lncRNAs), of which some were identified in platelets [52], constitute yet an additional group of regulatory non-protein-coding RNAs. LncRNAs can be subdivided into sense-, antisense-, intronic-, bidirectional-, and long intergenic ncRNAs and are characterized by a minimum length of >200 nt [87,88,89]. Importantly, lncRNAs have been shown to play important roles in several human diseases, especially in cancer [90,91,92,93]. In NSCLC, the expression of lncRNAs was analyzed in platelets in order to identify novel biomarkers of lung cancer. A selection of five lncRNAs was identified and further measurements revealed a differential expression of two lncRNAs, namely, Membrane-Associated Guanylate Kinase Inverted 2-Antisense RNA 3 (*MAGI2-AS3*) and Zinc Finger NFX1-Type Containing 1 Antisense RNA 1 (*ZFAS1*), in platelets of NSCLC patients compared to healthy subjects [94]. Both lncRNAs have been assigned tumor suppressive functions in human breast cancer [95,96,97], whereas *ZFAS1* seems to be a potent oncogenic lncRNA in other tumor types [98]. In platelets and plasma of NSCLC patients, *MAGI2-AS3* and *ZFAS1* were downregulated and their expression significantly correlated with tumor stage. In addition, *MAGI2-AS3* expression significantly correlated with lymph-node and distant metastasis and the authors suggested that both platelet-derived lncRNAs could be used as potential diagnostic biomarkers in NSCLC [94]. However, the molecular function of these and other lncRNAs in platelets is currently not well understood and it is tempting to speculate about a potential transfer of lncRNAs from platelets to diverse other cell-types to modulate expression programs and phenotypes.

#### 2.1.4. Circular RNAs (circRNAs)

Circular RNAs (circRNAs) belong to a group of highly stable non-coding RNA-species with largely unknown functional relevance in platelets. In general, circRNAs can act as sponges to sequester miRNA or RBPs. Moreover, they serve as scaffolds to mediate complex formation between specific enzymes and substrates and recruit proteins to specific locations (reviewed in [99]). CircRNA biogenesis involves the action of the spliceosome which connects the 5′ and downstream 3′ ends of exons within the same transcript resulting in a circular conformation which comes along with increased stability [100]. Interestingly, circRNAs were found to be abundant and highly enriched in human platelets as demonstrated by an analysis of publicly available RNA-Seq datasets of human platelets [50]. Here, the authors analyzed three total RNA-Seq datasets using a back-splice exon junction discovery pipeline and identified 33,829 distinct structures consistent with circRNAs. This number is about 15 times higher as reported for other cells and 24,632 (~73%) of the structures were also present in at least one other Encyclopedia of DNA Elements (ENCODE) RNA-Seq datasets previously mined [101]. In order to identify genes significantly enriched for circRNAs in platelets, the authors analyzed 8041 circRNA producing genes and identified 3162 significantly enriched. For the vast majority of enriched genes, the contribution of circRNA exons to total transcription was >60% in nucleated tissues but >80% in platelets, and for 15 genes it was 100% in all three samples. Thus, for some genes, only RNA-Seq reads derived from circRNA-producing exons could be detected within platelet samples. Importantly, circRNAs were also enriched in other anucleate cells, but not in cultured megakaryocytes and the authors suggested that this was mainly due to a higher degradation rate of linear RNAs compared to circRNAs in mature platelets leading to the strong enrichment of the latter over time. Intriguingly, extensive mRNA degradation could explain some conflicting results concerning the correlation between the platelet transcriptome and proteome [50]. Nevertheless, the biological relevance of platelet circRNAs, if any, remains unclear: do they modulate platelet functions or are they simply byproducts of megakaryocyte transcription and platelet mRNA degradation? Compelling evidence against a rather random role of circRNAs in platelets comes from the finding that they are differentially sorted into platelet-derived microvesicles via a specific, yet unknown mechanism [102]. The selective release of circRNAs might represent a novel way of transferring information as vesicles from platelets to recipient cells, e.g., endothelial cells thereby inducing inflammatory responses. Moreover, the same study could show that platelet circRNAs associated with protein complexes of distinct sizes, so called circRNPs, as demonstrated for six highly abundant circRNAs, including a platelet-specific circRNA, namely, *Plt-circR4* [102]. However, the circRNA-specific binding partners and the functional significance of these interactions remain to be clarified.

In summary, circRNAs are highly enriched in platelets, they associate with proteins and can be released from platelets. However, not much is known about their regulatory roles in or outside platelets. In addition, their clinical implications remain unclear and it would be interesting to know if the abundance and/or composition of platelet circRNAs varies depending on the age and health status of individual subjects.

#### 2.1.5. Viral RNAs

A fascinating aspect of platelets is the appearance of viral RNA in platelets and their ability to aid in viral replication and the spread of virus in infected individuals [103,104]. Although both DNA- and RNA-containing viruses associate with platelets, only RNA viruses are able to replicate, as platelets are incapable of DNA transcription due to their anucleate nature. A study on platelets isolated from human immunodeficiency virus (HIV)-infected patients on antiretroviral drug therapy (ART) revealed the occurrence of cytosolic HIV RNA and intact viral particles [105]. Noteworthy, replication competent viruses were only observed within closed vacuoles and not on the platelet surface. During the course of platelet elimination by tissue macrophages, HIV was transmitted from platelets to macrophages via phagocytosis. This process was prohibited by blocking platelet–macrophage interactions with the platelet-specific anti-integrin α_IIb_/β3 antibody abciximab, underpinning the fact that viral spread is supported by intercellular interactions. Most interestingly, a low CD4^+^ T cell count was predictive for the presence of HIV-containing platelets in patients receiving ART, which correlated with immunological failure in these individuals [105]. In contrast, a recent study found no correlation of HIV^+^ platelets with CD4^+^ T-cell count but with viral load and number of HIV^+^ platelets were significantly reduced after ART. This study additionally confirmed the ability of platelets to promote HIV viral spread, which was dependent on platelet activation and subsequent platelet-CD4^+^ T-cell complex formation [106]. Importantly, platelet-virus interactions are not unique to HIV, but many other viruses were shown to infect platelets. Platelets have been shown to be permissive to Dengue virus entry, further enabling translation of viral RNA, replication of the viral genome, and assembly of infectious virus. This study was further able to identify heparan sulfate proteoglycan (HSP) and dendritic cell-specific intercellular adhesion molecule-3-grabbing nonintegrin (DC-SIGN) as the major receptors for virus-platelet interactions [107]. Other viruses that have been shown to interact with platelets, mostly via surface integrins, and are able to enter these include different adenoviruses, influenza virus and encephalomyocarditis virus (reviewed in [103,104]).

Starting in December 2019, severe acute respiratory syndrome coronavirus 2 (SARS-CoV-2) infections became a global problem with unforeseen consequences. Some Corona Virus Disease 2019 (COVID-19) patients show hypercoagulation and thrombosis, which contributes to organ failure and worsened outcomes [108]. These clinical observations led to the hypothesis that platelets may play a role in COVID-19 progression. Protein or RNA expression of the main receptor for SARS-CoV-2 binding, angiotensin-converting enzyme 2 (ACE2), was not detected in platelets. Nevertheless, mRNA from the SARS-CoV-2 N1 gene was found in platelets of some COVID-19 patients suggesting an ACE2-independent entry of the virus into platelets [109,110]. In addition, platelets might be attracted by the loss of endothelial integrity caused by the SARS-CoV-2 entry into endothelial cells, which ultimately results in platelet activation and degranulation thereby leading to a cytokine storm. Thus, platelets might not only facilitate the dissemination of SARS-CoV-2 in infected individuals but might also contribute to hazardous thromboinflammation by the release of pro-inflammatory cytokines. Moreover, megakaryocytes from the lungs might be susceptible to SARS-CoV-2, thereby generating SARS-CoV-2 RNA-containing platelets with an altered transcriptome. RNA-Seq analysis of platelets isolated from severely ill COVID-19 patients compared to healthy donors displayed significant differences in RNA expression patterns associated with changes in platelet activation and aggregation [110]. Whether platelets constitute a target for COVID-19 therapy to alleviate the course of disease in severe cases will be an interesting topic for future research.

### 2.2. Sources of Platelet RNA

As described above, platelets contain a diverse collection of coding and non-coding as well as linear and circular RNAs, posing the question of the origins of these transcripts and the underlying molecular mechanisms, if any, that are responsible for the sorting of these RNAs into platelets. Intuitively, MKs seem to be the prime source of platelet RNAs and, indeed, MKs transcribe a wide set of mRNAs, which they pass on to their progeny in a highly regulated manner [111,112]. As proven by transcriptome analysis, platelets contain thousands of MK-derived mRNAs [45,113,114,115,116,117]. Careful analysis of RNA expression data on Matrix Metalloproteinase (*MMP*) and Tissue Inhibitor of Metalloproteinase (*TIMP*) mRNAs uncovered different proportions of expression profiles in platelets compared to their abundancy in MKs [118]. Excluding mRNA degradation or instability, Cecchetti et al. hypothesized that MKs differentially sort mRNAs into platelets. This hypothesis is supported by the observation that MKs expressed 10 out of 24 MMPs and three out of four TIMPs, yet several of these mRNAs were missing or barely detectable in platelets, including transcripts encoding *MMPs 2*, 9, 14, and *17* and *TIMP-3*. Moreover, the abundance of a transcript in MKs did not uniformly predict its expression level in platelets. For example, *TIMP-1* mRNA is expressed at high levels in both MKs and platelets while *TIMP-3* mRNA is abundantly expressed in MKs only, suggesting a regulated transfer of individual mRNAs [118]. However, the exact sorting mechanisms remain elusive, but it was suggested that MKs, similar to neurons, leverage RBPs that bind to certain nascent transcripts in the nucleus thereby forming a complex that translocates to the cytoplasm. Upon association with a kinesin motor, this complex is transported through the proplatelet by microtubules, and is ultimately delivered to the nascent platelet [119]. In fact, Cecchetti et al. could detect the mRNAs of a selected group of RBPs, which were previously shown to be involved in RNA localization, both in MKs and platelets [118]. However, the expression and localization of the corresponding proteins await further investigation.

In addition to MKs, platelets may also acquire RNA species by either direct or indirect interaction with surrounding cells in the circulation or in tissue microenvironments. In glioblastoma patients, platelets sequestered tumor-specific EGFR variant III (*EGFRvIII*) mRNA, which was also detected in corresponding plasma samples [94]. In platelets from NSCLC patients, Echinoderm Microtubule Associated Protein Like 4-Anaplastic Lymphoma Kinase (*EML4-ALK*) rearrangements were detected on transcript level. The transfer of this RNA was likely mediated via exosome shedding of cancer cells, thus confirming cross talk between platelets and tumor cells. Most interestingly, serial monitoring of *EML4-ALK* rearrangements in platelets showed a loss of detectable *EML4-ALK* rearrangements after clinical responds to crizotinib treatment [120].

Another intriguing, although likely indirect form of transfer of genetic information from cells to platelets might be emperipolesis, a process, where intact cells are present within the cytoplasm of another cell. Histological examination of bone marrow routinely identifies MKs, which have engulfed neutrophils or other hematopoietic cells. Hence, emperipolesis is a physiological process that can be increased in pathological conditions, e.g., in gray platelet syndrome or essential thrombocythemia [121,122]. Recent studies have shown that emperipolesis of intact neutrophils in MKs is mediated in part through the β2-integrin/ Intercellular Adhesion Molecule 1 (ICAM-1)/ezrin pathway. Neutrophil membranes merged with the DMS and thereby transferred membrane material to daughter platelets, which accelerated platelet production [123]. Intriguingly, some circulating platelets thus represent hybrids carrying both MK and neutrophil components. However, the mechanisms and functions of this conserved cellular phenomenon are largely unknown and whether emperipolesis is relevant for the transfer of RNA into platelets should be investigated in the future.

## 3. Role of Non-Coding RNAs in Megakaryopoiesis, Platelet Biogenesis and Function

Megakaryopoiesis, megakaryocyte maturation, as well as platelet formation, were shown to be highly complex processes that are regulated on multiple levels including epigenetic, transcriptional as well as post-transcriptional gene expression control mechanisms. Indeed, several miRNAs, which fine-tune gene expression at the post-transcriptional level, were shown to affect MK differentiation and platelet formation. For example, an inhibitory role for miR-155 during megakaryopoiesis was described, which is in line with the observed downregulation of this miRNA during the differentiation of CD34+ hematopoietic stem cells along the megakaryocyte lineage [124]. In contrast, miR-150 was shown to support normal megakaryocyte differentiation by targeting the transcription factor *MYB* [125,126]. Additionally, miR-130 targets the MAF BZIP Transcription Factor B (*MAFB*), which increases with terminal MK differentiation and induces activation of the Integrin Alpha 2b (*ITGA2B*) gene [127]. Very recently, miR-22 has been shown to promote megakaryocyte differentiation through repression of its target Growth Factor Independent 1 Transcriptional Repressor (*GFI1*) [128]. Ex vivo, megakaryocyte differentiation from hematopoietic stem/progenitor cells (HSPCs) and generation of platelets was also shown to be regulated by miRNAs. Specifically, miR-125b and miR-660 increased the percentage of megakaryocytes with high ploidy and upregulated platelet biogenesis compared to controls. In contrast, overexpression of the miR-23a/27a/24-2 cluster in HSPCs reduced colony formation associated with diminished megakaryocyte maturation and platelet production [129]. The importance of miRNAs in the regulation of development and function of MKs and platelets also becomes apparent upon MK-specific deletion of the ribonuclease *Dicer1* that is required for miRNA biogenesis. Knockdown of *Dicer1* in an in vivo mouse model led to reduced levels of miRNAs in platelets and hence to an altered platelet mRNA expression profile. The resulting phenotype was characterized by enhanced platelet reactivity and hemostatic function, presumably due to the upregulation of the fibrinogen receptor subunits integrin α_IIb_ and β_3_ on the platelet surface in the absence of a proper miRNA machinery [130]. Furthermore, a global approach to identify miRNAs and their respective targets in platelets detected Protein Kinase CAMP-Dependent Type II Regulatory Subunit Beta (*PRKAR2B*) as a miR-200b target, which functionally controlled platelet activation and altered levels of PRKAR2B led to impaired platelet reactivity [131]. Moreover, hyperreactive platelets were found to have elevated levels of Vesicle-Associated Membrane Protein 8 (*VAMP8*), a critical protein for granule release, which is a target of miR-96. Overexpression of miR-96 restored normal VAMP8 protein levels and thus confirmed the miR-96-VAMP8 interaction [132]. Importantly, alterations of miRNA expression patterns have been linked to malignancies related to the megakaryocytic lineage, including essential thrombocythemia (ET) [133]. In detail, it was shown that the expression of miR-9 and miR-490 significantly increased in platelets of ET patients, while others, including miR-409 and miR-126, were downregulated. MiRNA expression was correlated with platelet aggregation and expression of platelet surface receptors including CD63 and P-Selectin, suggesting that miRNA may play a role in ET, platelet maturation and function [133]. However, controversy exists as to what extent platelet-specific miRNAs are important for platelet biology itself. Some of the skepticism comes from genetic knockout studies in mice in which loss of miR-223, a highly abundant well-studied miRNA in human platelets [134], was dispensable for platelet production, both in terms of number and size as well as platelet functionality including overall life-span and surface expression of platelet adhesion receptors important for platelet activation like the adenosine diphosphate (ADP) receptor P2Y12 [135]. However, the lack of an overt phenotype might be due compensatory mechanisms as well as redundant targeting and functional rescue by other miRNAs. Moreover, lack of regulation of well-known miR-223 target transcripts might be explained by the lack of binding sites in the 3′-UTR of murine as compared to human genes as evidenced in the case of *P2Y12* [136].

Next to miRNAs, lncRNAs have also been implicated in the control of megakaryopoiesis. In general, lncRNAs can regulate several cellular processes including differentiation applying a broad range of epigenetic, transcriptional as well as post-transcriptional mechanisms [90,137]. For example, the expression of the human RNA Binding Motif Protein 15 (RBM15) was shown be positively regulated by its antisense lncRNA, *AS-RBM15*, during megakaryopoiesis [138]. Importantly, the RNA-binding protein RBM15 is known for its role in promoting terminal megakaryocyte differentiation through its impact on the alternative splicing of key transcription factors [139]. Intriguingly, RBM15 protein synthesis underlies a complex regulatory mechanism itself involving *AS-RBM15* and the transcription factor RUNX1 [138]. Specifically, *AS-RBM15*, which is transcribed in the opposite direction within exon 1 of RBM15, was shown to enhance 5′cap-dependent protein translation of RBM15. This might be achieved via putative complementary binding of exon 1 of *AS-RBM15* deep within the 5′-UTR of *RBM15* and its incorporation into the *RBM15* mRNA-containing polysomes. Both *AS-RBM15* and *RBM15* transcriptions were activated by the transcription factor RUNX1 and repressed by the leukemic fusion protein RUNX1-ETO, suggesting that *AS-RBM15* is not only important for megakaryocyte differentiation, but might also be implicated in leukemogenesis [138]. Additional, although indirect, evidence that indicated a functional role of lncRNAs in MKs is provided by a transcriptome analysis, which identified 1109 polyadenylated lncRNAs expressed in erythroblasts, megakaryocytes, and megakaryocyte-erythroid precursors of mice, and 594 lncRNAs expressed in human erythroblasts [140]. Importantly, it was recently shown that megakaryocyte-enriched protein-coding genes, but not erythroid ones, are “primed” in HSPCs, and that these genes are occupied by a group of seven transcription factors (TFs) that facilitate low-level expression in HSPCs [141]. Lineage-specific alterations in TF occupancy subsequently led to an activation of these genes during megakaryopoiesis and repression during erythropoiesis. Intriguingly, similar observations could be made for lncRNAs and the model that some megakaryocyte-enriched genes are specifically primed in HSPCs could be extended to lncRNA loci, emphasizing common regulatory mechanisms with coding genes [140]. However, the relevance of these lncRNAs for MK differentiation and function besides their regulation remains unclear and requires further investigation. In contrast, more direct evidence for the relevance of lncRNAs for MK and platelet function was recently presented in the context of type 2 diabetes. Here, platelet hyperaggregation and hypercoagulation are often observed in patients with type 2 diabetes (T2D) leading to an increased thrombogenic risk [142]. However, T2D patients frequently fail to respond to commonly used ADP receptor blocker such as clopidogrel and Cangrelor that are used as antiplatelet therapy thus leaving residual platelet reactivity [143]. Moreover, high activity of the ADP receptor P2Y12 is found in T2D patients, exposing such patients to a prothrombotic condition [144,145]. In this context, the non-coding Metallothionein 1 Pseudogene 3 (*MT1P3*) was found to be significantly upregulated in MKs from type 2 diabetes patients compared to healthy controls and, most importantly, had an impact on platelet activation and expression of the *P2Y12* by sponging miR-126. Of note, depletion of *MT1P3* by small interfering RNA (siRNA) reduced the expression of the ADP receptor, thereby inhibiting platelet activation and aggregation in a murine diabetes model [146].

In summary, small and long non-coding RNAs are crucial regulators of megakaryocyte and platelet biology, and changes in ncRNA expression are associated with alterations in megakaryopoiesis, platelet biogenesis and platelet function. However, our current understanding of the underlying molecular mechanisms is still very limited and much more research, especially on the role of lncRNAs, is needed.

## 4. Post-Transcriptional Gene Expression Control by RBPs and Its Role in Platelet Biogenesis and Function

### 4.1. General Aspects of Translational Control Mechanisms

**Figure 2:**
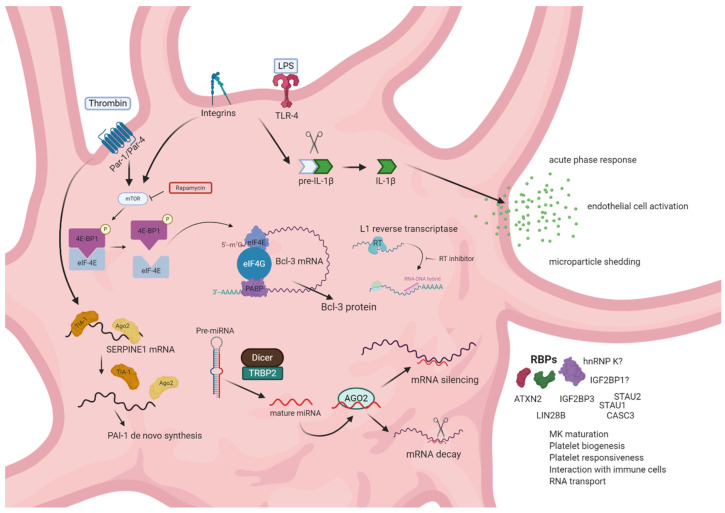
Posttranscriptional gene expression control in platelets. Receptor-mediated signaling may lead to splicing and translational events that are silenced in resting platelets. Lipopolysaccharide (LPS) binding to toll-like receptor 4 (TLR4) and integrin signaling induces the proteolytic processing of *pre-IL-1β* to mature interleukin *(IL)-1β*, resulting in pro-inflammatory signaling. Integrin- and thrombin receptor mediated platelet activation induces reorganization of the translation initiation complex and translation of certain mRNAs, including serpin family E member 1 (*SERPINE1*) and BCL3 transcription coactivator (*Bcl-3*), partially in an mechanistic target of rapamycin (mTOR)-dependent manner. Posttranscriptional gene expression control is also guided by miRNA-binding to the 3′-untranslated region (UTR) of target genes regulating RNA degradation or translation. An additional layer of posttranscriptional gene expression control is represented by RBPs responsible for megakaryocyte MK maturation, platelet biogenesis, platelet responsiveness and platelet interaction with immune cells as well as RNA transport from MKs to proplatelets. Lastly, posttranscriptional regulation is mediated by L1 reverse transcriptase activity, which induces formation of RNA–DNA hybrids, thereby regulating global protein synthesis.

Post-transcriptional control mechanisms are crucial for platelets to dynamically alter their proteome, phenotype and function (summarized in Figure 2). Newly formed, anucleate platelets seem to have a higher biosynthetic potential than older ones, indicating post-transcriptional regulatory circuits acting during the life cycle of platelets [147]. Post-transcriptional gene expression control is mainly achieved through miRNAs via binding to the 3′-UTR of their target transcript thereby regulating RNA degradation and protein translation as mentioned above. In addition to miRNAs, a second major class of post-transcriptional regulators consists of RBPs. RBPs recognize specific sequence or structure elements often present in the UTRs of their target RNAs. Interestingly, platelets were found to have, on average, much longer 3′-UTR compared to nucleated cells (1047 nt versus 492 nt, respectively), indicating an elongated binding region for miRNAs and RBPs thereby allowing potentially more complex regulation of translational and RNA stability [44].

Intriguingly, the key regulators of eukaryotic protein synthesis, i.e., eukaryotic translation initiation factor (eIF) 4E and eIF2α, are highly abundant in platelets [148]. Furthermore, key ribosomal components, including ribosomal protein S6 as well as 18S and 28S ribosomal RNAs (rRNAs), are present in platelets [112,115]. However, translation in resting platelets is generally prohibited or reduced via binding of eIF4E to the inhibitory protein 4E-binding protein 1 (4E-BP1), which prevents the assembly of the eIF4F initiation complex. Activation of the mechanistic target of rapamycin (mTOR) signaling pathway leads to phosphorylation of 4E-BP1 and to its dissociation from eIF4E, thereby enabling formation of the initiation complex and subsequent translation in platelets [114,149]. Of note, pharmacological inhibition of mTOR via rapamycin has controversial effects on platelet activity. While collagen-induced platelet aggregation was significantly reduced [150], platelet adhesion to endothelial cells was facilitated by rapamycin through the remodeling of the endothelial membrane [151]. Furthermore, a study that investigated the long-term effects of mTOR inhibitors in patients that received kidney transplants observed altered platelet functions. The side effects included reduced granule secretion and impaired platelet aggregation, because of biphasic time-dependent alterations in calcium homeostasis and function in platelets [152]. These findings can be, at least partially, explained by the observation that inhibition of mTOR activity using rapamycin reduced translational events of certain, but not all transcripts indicating specificity of mTOR signaling-dependent protein synthesis [153]. Importantly, these examples underscore the relevance of translational control in platelets. This is further supported by the observation that some proteins are constitutively translated in platelets, whereas the translation of others is regulated in a signal-dependent manner as it has been shown for BCL3 transcription coactivator (*Bcl-3*) and *IL-1β* [114,115,149,154]. Mechanistically, platelet integrins have been proposed to transmit outside-in signaling cascades thereby modulating signal-dependent translation in platelets. For example, while quiescent platelets harbor mRNA for the precursor IL-1β cytokine (pre-IL-1β) at the site of polysomes, its translation is induced upon β_3_ integrin signaling [115]. Moreover, integrin signaling can lead to a redistribution of eIF4E to mRNA-rich areas in aggregated platelets thereby initiating protein translation upon platelet activation [114]. Similarly, translation of *Bcl-3* mRNA is constitutively repressed in resting platelets. However, upon thrombin receptor-mediated activation of platelets, Bcl-3 protein is rapidly synthesized within minutes and the translation may be modulated via engagement of integrin α_IIb_β_3_ [155]. Rapamycin was shown to abrogate Bcl-3 translation indicating a regulatory role of mTOR in signal-dependent protein translation in platelets. Importantly, signal-dependent translation in platelets is not exclusively regulated via integrins. Early studies investigated the impact of highly polyunsaturated fatty acids of the n-3 family on the platelet antioxidant status and observed elevated enzyme activity of glutathione-dependent peroxidase (GPx), which counteracts oxidative stress. The enhanced enzyme activity could be explained by increased protein translation, since concomitant treatment with the protein synthesis inhibitor cycloheximide abolished the additional enzyme activity [156].

### 4.2. Role of RNA Binding Proteins (RBPs) in MKs and Platelets

Besides these examples of general mechanisms that contribute to translational control in platelets, also more specific regulations of individual mRNA by certain RBPs have been identified. Of note, more than 1500 human proteins have been annotated as RBPs based on experimental evidence and the presence of canonical RNA-binding domains (RBDs) [157]. RBPs typically use RBDs, such as the RNA recognition motif (RRM), hnRNP K homology domain (KH) or DEAD box helicase (DDX) domain, in order to bind to their target RNA and subsequently form ribonucleoprotein (RNP) complexes. However, additional RBPs have been identified lacking these characteristic RBDs [158]. Importantly, RBPs are crucial post-transcriptional regulators that are able to execute a plethora of control mechanisms, including regulation of RNA translation, splicing, transport, decay and editing [157]. Hence, RBPs could potentially contribute to multiple aspects of MK differentiation, platelet formation as well as platelet function. In line with this, a recent study revealed that the RBP ATAXIN2 (ATXN2) modulates the MK transcriptome and proteome and affects expression of platelet surface proteins [159]. ATXN2 has been implicated in regulating global mRNA stability and translation and directly binds to over 4000 transcripts as evaluated by photoactivatable-ribonucleoside-enhanced crosslinking and immunoprecipitation (PAR-CLIP) [160,161]. In human megakaryoblasts, ATXN2 was shown to associate with the poly(A)-binding protein (PABP) and the RNA helicase DDX6, and knockdown of ATXN2 in MEG-01 cells yielded 454 differentially expressed RNAs and 20 differentially expressed proteins [159]. However, the authors of this study did not identify direct RNA targets of ATXN2 in megakaryoblasts. Intriguingly, *Atxn2* knockout mice exhibited a decrease in type IV megakaryoid cells, and αIIbβ3 integrin-mediated platelet aggregation was impaired upon stimulation with phorbol myristate acetate (PMA) or with Aggretin A. This could be due to deregulated total expression of ITGB3 and aberrant surface receptor expression of CD31 in conjunction with C6orf25, Coagulation Factor II Thrombin Receptor (F2R) and VAV1 dysregulation in *Atxn2* knockout mice [159]. In addition to ATXN2, other RBPs have been implicated in megakaryopoiesis and MK maturation. As mentioned earlier, RBM15 was shown to play a role in megakaryocytic differentiation of hematopoietic stem cells acting in concert with protein arginine methyltransferase 1 (PRMT1) which methylates RBM15 and thereby regulates its stability via the E3 ubiquitin ligase CNOT4 [139,162]. RBM15 was shown to bind to intronic sequences and interact with the Splicing Factor 3b Subunit 1 (SF3B1) thereby regulating alternative splicing of genes that are important for megakaryopoiesis such as *GATA1*, *RUNX1*, T-Cell Acute Lymphocytic Leukemia 1 (*TAL1*) and *c-MPL* [139]. Another RBP that may influence megakaryocyte differentiation is Insulin-like Growth Factor 2 mRNA-binding protein 1 (IGF2BP1). IGF2BP1 has been found to target the ETS Variant Transcription Factor 6 *(ETV6)/RUNX1* fusion transcript and potentially regulates its stability in acute lymphatic leukemia (ALL) [163]. However, it is currently unknown, if IGF2BP1 modulates *RUNX1* expression in HSCs or megakaryocytes. However, it could be shown that another member of the IGF2BP family regulates the human fetal–adult megakaryocyte transition [164]. In detail, the oncofetal IGF2BP3 is expressed significantly higher in neonatal hematopoietic cells, including neonatal megakaryocytes, but is completely absent in its adult counterparts. High expression of IGF2BP3 restricted megakaryocyte morphogenesis and polyploidy by inhibiting the positive transcription elongation factor b (P-TEFb) kinase complex by binding and stabilization of the *7SK snRNA*. Consequently, high IGF2BP3 expression in neonatal megakaryocytes affected platelet function leading to hyporesponsiveness in full-term and premature neonates, which could lead to thrombocytopenia and bleeding commonly observed in premature neonates [164]. Another example of an oncofetal RBP with a role in regulating MK and platelet function is LIN28B, which is highly expressed in fetal, but not in adult megakaryocytes. High LIN28B expression in fetal megakaryocytes negatively regulated surface P-Selectin expression in fetal platelets, which influenced their interaction with neutrophils in vitro and in vivo [165]. Hence, differential RBP expression in developing MKs as well as old and young platelets may play a crucial role in regulating platelet function. In addition, post-transcriptional regulation by RBPs might also be vital in regulating platelet production and release from MKs, which includes cytoskeletal rearrangements. Myosin heavy chain 9 (MYH9) is a major non-muscle myosin expressed in MKs and platelets, associates with the actin cytoskeleton and enables morphogenesis. Mutations in or knockout of *MYH9* is linked to a group of platelet disorders leading to macro-thrombocytopenia, prolonged bleeding and clot retraction deficiency [166]. In erythropoiesis, MYH9 is crucial for erythroid cell enucleation. This process was shown to depend on the activity of heterogeneous nuclear ribonucleoprotein K (hnRNP K), an RBP that inhibits translation of *MYH9* mRNA [167]. It would be interesting to evaluate the effects of hnRNP K on *MYH9*-dependent megakaryocyte maturation and platelet biogenesis, which has not been investigated so far.

In addition to regulating stability and translation of mRNAs, RBPs can also be involved in the localization and transport of their target RNAs from megakaryocytes to platelets, which is suggested to occur in a controlled rather than random manner as mentioned above [118]. While the exact sorting mechanisms remain elusive, MKs and platelets were shown to contain mRNAs encoding for RBPs that were previously implicated in mRNA transport processes including Cancer Susceptibility Candidate Gene 3 (CASC3), Staufen Double-Stranded RNA Binding Protein 1 and 2 (STAU1 and STAU2) as well as EIF4A3 [118,168]. Hence, these RBPs might be expressed in MKs and platelets and may play a role in the regulated transport of coding and non-coding transcripts into platelets [169].

Consequently, RBPs have also been detected within platelets themselves where they modulate transcript stability and translation efficiency. Among the identified RBPs are T-cell internal antigen-1 (TIA-I), TIA-I-related protein (TIA-R), and ELAV like RNA binding protein 1 (ELAVL1, HuR) [112]. TIA-1 has been implicated in alternative splicing regulation of various pre-mRNAs and was shown to suppress translation [170]. Upon platelet activation by thrombin, TIA-I dissociates from the Serpin Family E Member 1 (*SERPINE1*) mRNA, thereby lifting repressive effects and allowing translation and de novo synthesis of Serpine1 protein, also known as Plasminogen Activator Inhibitor 1 (PAI-1). Similar effects were found for Ago2, where dissociation of Ago2 protein upon thrombin stimulation of platelets was found for *SERPINE1* mRNA whereas Ago2/miRNA complexes or associations of Ago2 with other mRNAs known to be translated upon platelet activation, including Prostaglandin-Endoperoxide Synthase 1 (*PTGS1*) and *ITGB3* mRNAs, were not affected. On the other hand, association of *SERPINE1* mRNA with the RBP HuR did not change upon platelet activation, which might indicate that HuR acts as a stabilizing factor rather than a translational regulator under these conditions [171]. Overall, these findings implicate some degree of specificity for the regulation of individual platelet mRNAs during platelet stimulation, which is likely distinct following platelet activation by other known platelet activating factors besides thrombin.

Finally, yet importantly, it is worth mentioning that not only miRNAs and RBPs could regulate protein synthesis in MKs and platelets, but also other post-transcriptional regulators might exist. Indeed, Schwertz et al. demonstrated that translational events in platelets could be controlled by endogenous long interspersed nuclear element-1 (LINE-1) reverse transcriptase activity (eRT) [172]. Intriguingly, inhibition of eRT in vitro in isolated platelets from healthy individuals or in people with HIV treated with RT inhibitors enhanced global protein synthesis. Moreover, platelet activation was induced promoting a pro-thrombotic functional response, which was likely mediated by the generation of RNA-DNA hybrids. These results present a novel, previously unrecognized translational regulatory mechanism, which could have clinical implications for HIV patients that are at an increased risk of thrombosis, which might be related to RT inhibitor-based antiretroviral therapies [173].

In summary, post-transcriptional regulatory pathways are crucial for rapid adaptations to changing environmental properties upon platelet activation. RBPs expressed in megakaryocytes and platelets are critical for megakaryocyte differentiation, maturation, and platelet genesis. Moreover, RBPs might be important transport factors mediating the sorting of RNAs from megakaryocytes into platelets and thereby influence signaling pathways in circulating platelets. However, several of the underlying mechanistic details are not well understood so far, which warrants further in-depth investigations in the future.

## 5. Conclusions

Platelets contain a plethora of RNAs that are inherited from megakaryocytes or taken up from interacting cells or microorganisms. The functions of these transcripts, whether they are crucial for platelet function per se independent of their role in megakaryocytes and the mechanisms that control their sorting and uptake as well as their constitutive or signal-dependent translation and decay, are just beginning to be resolved. Studying the contribution of RBPs to these processes will allow us to gain a deeper understanding of the complex networks underlying megakaryopoiesis, platelet biogenesis and function.

## Abbreviations

ACE2	angiotensin I converting enzyme 2
ADP	adenosine diphosphate
Ago2	argonaute RISC catalytic component 2
ALL	acute lymphoblastic leukemia
ART	antiretroviral drug therapy
AS-RBM15	RBM15 antisense RNA 1
ATXN2	ataxin 2
Bcl-3	BCL3 transcription coactivator
CASC3	Cancer Susceptibility Candidate Gene 3
circRNA	circular RNA
circRNP	circRNA-protein complex
c-MPL	cellular myeloproliferative leukemia virus
CNOT4	CCR4-NOT transcription complex subunit 4
COVID-19	corona virus induced disease 19
COX-2	cyclooxygenase 2
DDX6	DEAD-box helicase 6
DGCR8	DiGeorge syndrome critical region 8
DMS	demarcation membrane system
EPB41L3	Erythrocyte Membrane Protein Band 4.1-like 3
EFNA1	Ephrin A1
EGFR	Epidermal Growth Factor Receptor
eIF	eukaryotic translation initiation factor
EML4-ALK	Echinoderm Microtubule Associated Protein Like 4-Anaplastic Lymphoma Kinase
ER	endoplasmic reticulum
eRT	endogenous reverse transcriptase
ETO	RUNX1 partner transcriptional co-repressor 1
FBXW7	F-Box and WD Repeat Domain Containing 7
FXI	coagulation factor XI
GATA1	GATA binding protein 1
GFI1	Growth Factor Independent 1 Transcriptional Repressor
GPx	glutathione-dependent peroxidase
HER2	Human Epidermal Growth Factor Receptor 2
HIV	human immunodeficiency virus
hnRNP K	heterogeneous nuclear ribonucleoprotein K
HSC	hematopoietic stem cell
HuR/ELAVL1	ELAV like RNA binding protein 1
ICAM	intercellular adhesion molecule
IGF1R	Insulin-like Growth Factor 1 Receptor
IGF2BP1	Insulin-like growth factor 2 mRNA binding protein 1
IGF2BP3	Insulin-like growth factor 3 mRNA binding protein 3
IL-1β	interleukin 1β
ITGA2B	Integrin Alpha 2b
ITGB3	integrin subunit beta 3
KRAS	Kirsten Rat Sarcoma Viral Oncogene Homolog
LIN28B	lin-28 homolog B
LINE-1	long interspersed nuclear element-1
lncRNA	long non-coding RNA
LPS	lipopolysaccharide
m^7^G	7-methylguanosine
MAFB	MAF bZIP transcription factor B
MAGI2-AS3	MAGI2 antisense RNA 3
MET	Mesenchymal Epithelial Transition tyrosine kinase
miRNA	micro RNA
MK	megakaryocyte
MMP	matrix metallopeptidase
MPL	MPL proto-oncogene, thrombopoietin receptor
mRNA	messenger RNA
MT1P3	metallothionein 1 pseudogene 3
mtDNA	mitochondrial DNA
mTOR	mechanistic target of rapamycin kinase
MV	microvesicle
MYH9	myosin heavy chain 9
NGS	next generation sequencing
NSCLC	non-small-cell lung carcinoma
nt	nucleotide
P2Y12	purinergic receptor P2Y12
PABP	polyA binding protein
PAR-CLIP	photoactivatable-ribonucleoside-enhanced crosslinking and immunoprecipitation
PIK3CA	Phosphatidylinositol-4,5-Bisphosphate 3-Kinase Catalytic Subunit Alpha
PMA	peroneal muscular atrophy
PRKAR2B	protein kinase cAMP-dependent type II regulatory subunit beta
PRMT1	protein arginine methyltransferase 1
P-TEFb	positive transcription elongation factor b
PTGS1	prostaglandin-endoperoxide synthase 1
RBD	RNA-binding domains
RBM15	RNA Binding Motif Protein 15
RBP	RNA binding protein
RNA-Seq	RNA sequencing
RRM	RNA recognition motif
rRNA	ribosomal RNA
RUNX1	RUNX family transcription factor 1
SARS-CoV-2	severe acute respiratory syndrome coronavirus 2
SERPINE1	serpin family E member 1
SF3B1	splicing factor 3b subunit 1
siRNA	small interfering RNA
snRNA	small nuclear RNA
STAU1/2	Staufen Double-Stranded RNA Binding Protein 1 and 2
T2D	type 2 diabetes
TAL1	TAL bHLH transcription factor 1
TARBP2	Trans-Activation Responsive RNA-Binding Protein 2
TF	tissue factor
THBS-1	thrombospondin 1
TIA-I	TIA1 cytotoxic granule associated RNA binding protein
TIMP	TIMP metallopeptidase inhibitor
TLR4	Toll like receptor 4
TPO	thrombopoietin
UTR	untranslated region
VAMP8	vesicle associated membrane protein 8
ZFAS1	ZNFX1 antisense RNA 1

## References

[1] Mason K.D., Carpinelli M.R., Fletcher J.I., Collinge J.E., Hilton A.A., Ellis S., Kelly P.N., Ekert P.G., Metcalf D., Roberts A.W. (2007). Programmed anuclear cell death delimits platelet life span. Cell.

[2] Leeksma C.H., Cohen J.A. (1955). Determination of the life of human blood platelets using labelled diisopropylfluorophosphanate. Nature.

[3] George J.N. (2000). Platelets. Lancet.

[4] Franco A.T., Corken A., Ware J. (2015). Platelets at the interface of thrombosis, inflammation, and cancer. Blood.

[5] Boilard E., Nigrovic P.A., Larabee K., Watts G.F., Coblyn J.S., Weinblatt M.E., Massarotti E.M., Remold-O’Donnell E., Farndale R.W., Ware J. (2010). Platelets amplify inflammation in arthritis via collagen-dependent microparticle production. Science.

[6] Santilli F., Simeone P., Liani R., Davi G. (2015). Platelets and diabetes mellitus. Prostaglandins Other Lipid Mediat..

[7] Kroll M.H., Afshar-Kharghan V. (2012). Platelets in pulmonary vascular physiology and pathology. Pulm. Circ..

[8] Catricala S., Torti M., Ricevuti G. (2012). Alzheimer disease and platelets: Hows that relevant. Immun. Ageing.

[9] Willoughby S., Holmes A., Loscalzo J. (2002). Platelets and cardiovascular disease. Eur. J. Cardiovasc. Nurs..

[10] Gay L.J., Felding-Habermann B. (2011). Contribution of platelets to tumour metastasis. Nat. Rev. Cancer.

[11] Haemmerle M., Stone R.L., Menter D.G., Afshar-Kharghan V., Sood A.K. (2018). The Platelet Lifeline to Cancer: Challenges and Opportunities. Cancer Cell.

[12] Machlus K.R., Italiano J.E. (2013). The incredible journey: From megakaryocyte development to platelet formation. J. Cell Biol..

[13] Bartley T.D., Bogenberger J., Hunt P., Li Y.S., Lu H.S., Martin F., Chang M.S., Samal B., Nichol J.L., Swift S. (1994). Identification and cloning of a megakaryocyte growth and development factor that is a ligand for the cytokine receptor Mpl. Cell.

[14] Kaushansky K., Lok S., Holly R.D., Broudy V.C., Lin N., Bailey M.C., Forstrom J.W., Buddle M.M., Oort P.J., Hagen F.S. (1994). Promotion of megakaryocyte progenitor expansion and differentiation by the c-Mpl ligand thrombopoietin. Nature.

[15] Wendling F., Maraskovsky E., Debili N., Florindo C., Teepe M., Titeux M., Methia N., Breton-Gorius J., Cosman D., Vainchenker W. (1994). cMpl ligand is a humoral regulator of megakaryocytopoiesis. Nature.

[16] Alexander W.S., Roberts A.W., Nicola N.A., Li R., Metcalf D. (1996). Deficiencies in progenitor cells of multiple hematopoietic lineages and defective megakaryocytopoiesis in mice lacking the thrombopoietic receptor c-Mpl. Blood.

[17] de Sauvage F.J., Carver-Moore K., Luoh S.M., Ryan A., Dowd M., Eaton D.L., Moore M.W. (1996). Physiological regulation of early and late stages of megakaryocytopoiesis by thrombopoietin. J. Exp. Med..

[18] Gurney A.L., Carver-Moore K., de Sauvage F.J., Moore M.W. (1994). Thrombocytopenia in c-mpl-deficient mice. Science.

[19] Bunting S., Widmer R., Lipari T., Rangell L., Steinmetz H., Carver-Moore K., Moore M.W., Keller G.A., de Sauvage F.J. (1997). Normal platelets and megakaryocytes are produced in vivo in the absence of thrombopoietin. Blood.

[20] Carver-Moore K., Broxmeyer H.E., Luoh S.M., Cooper S., Peng J., Burstein S.A., Moore M.W., de Sauvage F.J. (1996). Low levels of erythroid and myeloid progenitors in thrombopoietin-and c-mpl-deficient mice. Blood.

[21] Patel S.R., Hartwig J.H., Italiano J.E. (2005). The biogenesis of platelets from megakaryocyte proplatelets. J. Clin. Investig..

[22] Zimmet J., Ravid K. (2000). Polyploidy: Occurrence in nature, mechanisms, and significance for the megakaryocyte-platelet system. Exp. Hematol..

[23] Lordier L., Jalil A., Aurade F., Larbret F., Larghero J., Debili N., Vainchenker W., Chang Y. (2008). Megakaryocyte endomitosis is a failure of late cytokinesis related to defects in the contractile ring and Rho/Rock signaling. Blood.

[24] Zhang Y., Wang Z., Liu D.X., Pagano M., Ravid K. (1998). Ubiquitin-dependent degradation of cyclin B is accelerated in polyploid megakaryocytes. J. Biol. Chem..

[25] Zhang Y., Wang Z., Ravid K. (1996). The cell cycle in polyploid megakaryocytes is associated with reduced activity of cyclin B1-dependent cdc2 kinase. J. Biol. Chem..

[26] Eckly A., Heijnen H., Pertuy F., Geerts W., Proamer F., Rinckel J.Y., Leon C., Lanza F., Gachet C. (2014). Biogenesis of the demarcation membrane system (DMS) in megakaryocytes. Blood.

[27] Scurfield G., Radley J.M. (1981). Aspects of platelet formation and release. Am. J. Hematol..

[28] Schwertz H., Koster S., Kahr W.H., Michetti N., Kraemer B.F., Weitz D.A., Blaylock R.C., Kraiss L.W., Greinacher A., Zimmerman G.A. (2010). Anucleate platelets generate progeny. Blood.

[29] Lefrancais E., Ortiz-Munoz G., Caudrillier A., Mallavia B., Liu F., Sayah D.M., Thornton E.E., Headley M.B., David T., Coughlin S.R. (2017). The lung is a site of platelet biogenesis and a reservoir for haematopoietic progenitors. Nature.

[30] Thon J.N., Montalvo A., Patel-Hett S., Devine M.T., Richardson J.L., Ehrlicher A., Larson M.K., Hoffmeister K., Hartwig J.H., Italiano J.E. (2010). Cytoskeletal mechanics of proplatelet maturation and platelet release. J. Cell Biol..

[31] Kaushansky K. (2006). Lineage-specific hematopoietic growth factors. N. Engl. J. Med..

[32] Di Buduo C.A., Kaplan D.L., Balduini A. (2017). In vitro generation of platelets: Where do we stand?. Transfus. Clin. Biol..

[33] Mookerjee S., Foster H.R., Waller A.K., Ghevaert C.J. (2020). In vitro-derived platelets: The challenges we will have to face to assess quality and safety. Platelets.

[34] Strassel C., Gachet C., Lanza F. (2018). On the way to in vitro platelet production. Transfus. Clin. Biol..

[35] Weyrich A.S., Schwertz H., Kraiss L.W., Zimmerman G.A. (2009). Protein synthesis by platelets: Historical and new perspectives. J. Thromb. Haemost..

[36] Versteeg H.H., Heemskerk J.W., Levi M., Reitsma P.H. (2013). New fundamentals in hemostasis. Physiol. Rev..

[37] Semple J.W., Italiano J.E., Freedman J. (2011). Platelets and the immune continuum. Nat. Rev. Immunol..

[38] Sharda A., Flaumenhaft R. (2018). The life cycle of platelet granules. F1000Res.

[39] Behnke O. (1989). Coated pits and vesicles transfer plasma components to platelet granules. Thromb. Haemost..

[40] Hanby H.A., Bao J., Noh J.Y., Jarocha D., Poncz M., Weiss M.J., Marks M.S. (2017). Platelet dense granules begin to selectively accumulate mepacrine during proplatelet formation. Blood Adv..

[41] Italiano J.E., Richardson J.L., Patel-Hett S., Battinelli E., Zaslavsky A., Short S., Ryeom S., Folkman J., Klement G.L. (2008). Angiogenesis is regulated by a novel mechanism: Pro- and antiangiogenic proteins are organized into separate platelet alpha granules and differentially released. Blood.

[42] Sehgal S., Storrie B. (2007). Evidence that differential packaging of the major platelet granule proteins von Willebrand factor and fibrinogen can support their differential release. J. Thromb. Haemost..

[43] Denis M.M., Tolley N.D., Bunting M., Schwertz H., Jiang H., Lindemann S., Yost C.C., Rubner F.J., Albertine K.H., Swoboda K.J. (2005). Escaping the nuclear confines: Signal-dependent pre-mRNA splicing in anucleate platelets. Cell.

[44] Dittrich M., Birschmann I., Pfrang J., Herterich S., Smolenski A., Walter U., Dandekar T. (2006). Analysis of SAGE data in human platelets: Features of the transcriptome in an anucleate cell. Thromb. Haemost..

[45] Gnatenko D.V., Dunn J.J., McCorkle S.R., Weissmann D., Perrotta P.L., Bahou W.F. (2003). Transcript profiling of human platelets using microarray and serial analysis of gene expression. Blood.

[46] Harrison P., Goodall A.H. (2008). “Message in the platelet“—More than just vestigial mRNA!. Platelets.

[47] Rowley J.W., Oler A.J., Tolley N.D., Hunter B.N., Low E.N., Nix D.A., Yost C.C., Zimmerman G.A., Weyrich A.S. (2011). Genome-wide RNA-seq analysis of human and mouse platelet transcriptomes. Blood.

[48] Schubert S., Weyrich A.S., Rowley J.W. (2014). A tour through the transcriptional landscape of platelets. Blood.

[49] Landry P., Plante I., Ouellet D.L., Perron M.P., Rousseau G., Provost P. (2009). Existence of a microRNA pathway in anucleate platelets. Nat. Struct. Mol. Biol..

[50] Alhasan A.A., Izuogu O.G., Al-Balool H.H., Steyn J.S., Evans A., Colzani M., Ghevaert C., Mountford J.C., Marenah L., Elliott D.J. (2016). Circular RNA enrichment in platelets is a signature of transcriptome degradation. Blood.

[51] Angenieux C., Maitre B., Eckly A., Lanza F., Gachet C., de la Salle H. (2016). Time-Dependent Decay of mRNA and Ribosomal RNA during Platelet Aging and Its Correlation with Translation Activity. PLoS ONE.

[52] Bray P.F., McKenzie S.E., Edelstein L.C., Nagalla S., Delgrosso K., Ertel A., Kupper J., Jing Y., Londin E., Loher P. (2013). The complex transcriptional landscape of the anucleate human platelet. BMC Genom..

[53] McRedmond J.P., Park S.D., Reilly D.F., Coppinger J.A., Maguire P.B., Shields D.C., Fitzgerald D.J. (2004). Integration of proteomics and genomics in platelets: A profile of platelet proteins and platelet-specific genes. Mol. Cell Proteom..

[54] Kissopoulou A., Jonasson J., Lindahl T.L., Osman A. (2013). Next generation sequencing analysis of human platelet PolyA+ mRNAs and rRNA-depleted total RNA. PLoS ONE.

[55] Mortazavi A., Williams B.A., McCue K., Schaeffer L., Wold B. (2008). Mapping and quantifying mammalian transcriptomes by RNA-Seq. Nat. Methods.

[56] Costa V., Angelini C., De Feis I., Ciccodicola A. (2010). Uncovering the complexity of transcriptomes with RNA-Seq. J. Biomed. Biotechnol..

[57] Zucker M., Hauschner H., Seligsohn U., Rosenberg N. (2018). Platelet factor XI: Intracellular localization and mRNA splicing following platelet activation. Blood Cells Mol. Dis..

[58] Schwertz H., Tolley N.D., Foulks J.M., Denis M.M., Risenmay B.W., Buerke M., Tilley R.E., Rondina M.T., Harris E.M., Kraiss L.W. (2006). Signal-dependent splicing of tissue factor pre-mRNA modulates the thrombogenicity of human platelets. J. Exp. Med..

[59] Brown G.T., McIntyre T.M. (2011). Lipopolysaccharide signaling without a nucleus: Kinase cascades stimulate platelet shedding of proinflammatory IL-1beta-rich microparticles. J. Immunol..

[60] Shashkin P.N., Brown G.T., Ghosh A., Marathe G.K., McIntyre T.M. (2008). Lipopolysaccharide is a direct agonist for platelet RNA splicing. J. Immunol..

[61] Londin E.R., Hatzimichael E., Loher P., Edelstein L., Shaw C., Delgrosso K., Fortina P., Bray P.F., McKenzie S.E., Rigoutsos I. (2014). The human platelet: Strong transcriptome correlations among individuals associate weakly with the platelet proteome. Biol. Direct..

[62] Rowley J.W., Weyrich A.S. (2013). Coordinate expression of transcripts and proteins in platelets. Blood.

[63] Best M.G., In ’t Veld S., Sol N., Wurdinger T. (2019). RNA sequencing and swarm intelligence-enhanced classification algorithm development for blood-based disease diagnostics using spliced blood platelet RNA. Nat. Protoc..

[64] Best M.G., Sol N., Kooi I., Tannous J., Westerman B.A., Rustenburg F., Schellen P., Verschueren H., Post E., Koster J. (2015). RNA-Seq of Tumor-Educated Platelets Enables Blood-Based Pan-Cancer, Multiclass, and Molecular Pathway Cancer Diagnostics. Cancer Cell.

[65] Best M.G., Vancura A., Wurdinger T. (2017). Platelet RNA as a circulating biomarker trove for cancer diagnostics. J. Thromb. Haemost..

[66] Best M.G., Wesseling P., Wurdinger T. (2018). Tumor-Educated Platelets as a Noninvasive Biomarker Source for Cancer Detection and Progression Monitoring. Cancer Res..

[67] Lieben L. (2015). Cancer genetics: RNA-seq for blood-based pan-cancer diagnostics. Nat. Rev. Genet..

[68] Bartel D.P. (2004). MicroRNAs: Genomics, biogenesis, mechanism, and function. Cell.

[69] Pichler M., Calin G.A. (2015). MicroRNAs in cancer: From developmental genes in worms to their clinical application in patients. Br. J. Cancer.

[70] Garzon R., Pichiorri F., Palumbo T., Iuliano R., Cimmino A., Aqeilan R., Volinia S., Bhatt D., Alder H., Marcucci G. (2006). MicroRNA fingerprints during human megakaryocytopoiesis. Proc. Natl. Acad. Sci. USA.

[71] Ple H., Landry P., Benham A., Coarfa C., Gunaratne P.H., Provost P. (2012). The repertoire and features of human platelet microRNAs. PLoS ONE.

[72] Provost P. (2017). The clinical significance of platelet microparticle-associated microRNAs. Clin. Chem. Lab. Med..

[73] Lee Y., Ahn C., Han J., Choi H., Kim J., Yim J., Lee J., Provost P., Radmark O., Kim S. (2003). The nuclear RNase III Drosha initiates microRNA processing. Nature.

[74] Denli A.M., Tops B.B., Plasterk R.H., Ketting R.F., Hannon G.J. (2004). Processing of primary microRNAs by the Microprocessor complex. Nature.

[75] Gregory R.I., Yan K.P., Amuthan G., Chendrimada T., Doratotaj B., Cooch N., Shiekhattar R. (2004). The Microprocessor complex mediates the genesis of microRNAs. Nature.

[76] Bernstein E., Caudy A.A., Hammond S.M., Hannon G.J. (2001). Role for a bidentate ribonuclease in the initiation step of RNA interference. Nature.

[77] Winter J., Jung S., Keller S., Gregory R.I., Diederichs S. (2009). Many roads to maturity: microRNA biogenesis pathways and their regulation. Nat. Cell Biol..

[78] Laffont B., Corduan A., Ple H., Duchez A.C., Cloutier N., Boilard E., Provost P. (2013). Activated platelets can deliver mRNA regulatory Ago2*microRNA complexes to endothelial cells via microparticles. Blood.

[79] Pan Y., Liang H., Liu H., Li D., Chen X., Li L., Zhang C.Y., Zen K. (2014). Platelet-secreted microRNA-223 promotes endothelial cell apoptosis induced by advanced glycation end products via targeting the insulin-like growth factor 1 receptor. J. Immunol..

[80] Gidlof O., van der Brug M., Ohman J., Gilje P., Olde B., Wahlestedt C., Erlinge D. (2013). Platelets activated during myocardial infarction release functional miRNA, which can be taken up by endothelial cells and regulate ICAM1 expression. Blood.

[81] Anene C., Graham A.M., Boyne J., Roberts W. (2018). Platelet microparticle delivered microRNA-Let-7a promotes the angiogenic switch. Biochim. Biophys. Acta Mol. Basis Dis..

[82] Liang H., Yan X., Pan Y., Wang Y., Wang N., Li L., Liu Y., Chen X., Zhang C.Y., Gu H. (2015). MicroRNA-223 delivered by platelet-derived microvesicles promotes lung cancer cell invasion via targeting tumor suppressor EPB41L3. Mol. Cancer.

[83] Willeit P., Zampetaki A., Dudek K., Kaudewitz D., King A., Kirkby N.S., Crosby-Nwaobi R., Prokopi M., Drozdov I., Langley S.R. (2013). Circulating microRNAs as novel biomarkers for platelet activation. Circ. Res..

[84] Zampetaki A., Kiechl S., Drozdov I., Willeit P., Mayr U., Prokopi M., Mayr A., Weger S., Oberhollenzer F., Bonora E. (2010). Plasma microRNA profiling reveals loss of endothelial miR-126 and other microRNAs in type 2 diabetes. Circ. Res..

[85] Zampetaki A., Willeit P., Tilling L., Drozdov I., Prokopi M., Renard J.M., Mayr A., Weger S., Schett G., Shah A. (2012). Prospective study on circulating MicroRNAs and risk of myocardial infarction. J. Am. Coll. Cardiol..

[86] Krammer T.L., Mayr M., Hackl M. (2020). microRNAs as promising biomarkers of platelet activity in antiplatelet therapy monitoring. Int. J. Mol. Sci..

[87] Mercer T.R., Dinger M.E., Mattick J.S. (2009). Long non-coding RNAs: Insights into functions. Nat. Rev. Genet..

[88] Quinn J.J., Chang H.Y. (2016). Unique features of long non-coding RNA biogenesis and function. Nat. Rev. Genet..

[89] Zhao Y., Teng H., Yao F., Yap S., Sun Y., Ma L. (2020). Challenges and Strategies in Ascribing Functions to Long Noncoding RNAs. Cancers (Basel).

[90] Gutschner T., Diederichs S. (2012). The hallmarks of cancer: A long non-coding RNA point of view. RNA Biol..

[91] Dorn A., Glass M., Neu C.T., Heydel B., Huttelmaier S., Gutschner T., Haemmerle M. (2020). LINC00261 Is Differentially Expressed in Pancreatic Cancer Subtypes and Regulates a Pro-Epithelial Cell Identity. Cancers (Basel).

[92] Gutschner T., Hammerle M., Eissmann M., Hsu J., Kim Y., Hung G., Revenko A., Arun G., Stentrup M., Gross M. (2013). The noncoding RNA MALAT1 is a critical regulator of the metastasis phenotype of lung cancer cells. Cancer Res..

[93] Sparber P., Filatova A., Khantemirova M., Skoblov M. (2019). The role of long non-coding RNAs in the pathogenesis of hereditary diseases. BMC Med. Genom..

[94] Luo C.L., Xu Z.G., Chen H., Ji J., Wang Y.H., Hu W., Wang K., Zhang W.W., Yuan C.H., Wang F.B. (2018). LncRNAs and EGFRvIII sequestered in TEPs enable blood-based NSCLC diagnosis. Cancer Manag. Res..

[95] Fan S., Fan C., Liu N., Huang K., Fang X., Wang K. (2018). Downregulation of the long non-coding RNA ZFAS1 is associated with cell proliferation, migration and invasion in breast cancer. Mol. Med. Rep..

[96] Xu X., Yuan X., Ni J., Guo J., Gao Y., Yin W., Li F., Wei L., Zhang J. (2020). MAGI2-AS3 inhibits breast cancer by downregulating DNA methylation of MAGI2. J. Cell. Physiol..

[97] Yang Y., Yang H., Xu M., Zhang H., Sun M., Mu P., Dong T., Du S., Liu K. (2018). Long non-coding RNA (lncRNA) MAGI2-AS3 inhibits breast cancer cell growth by targeting the Fas/FasL signalling pathway. Hum. Cell.

[98] Dong D., Mu Z., Zhao C., Sun M. (2018). ZFAS1: A novel tumor-related long non-coding RNA. Cancer Cell Int..

[99] Kristensen L.S., Andersen M.S., Stagsted L.V.W., Ebbesen K.K., Hansen T.B., Kjems J. (2019). The biogenesis, biology and characterization of circular RNAs. Nat. Rev. Genet..

[100] Cocquerelle C., Mascrez B., Hetuin D., Bailleul B. (1993). Mis-splicing yields circular RNA molecules. FASEB J..

[101] Salzman J., Chen R.E., Olsen M.N., Wang P.L., Brown P.O. (2013). Cell-type specific features of circular RNA expression. PLoS Genet..

[102] Preusser C., Hung L.H., Schneider T., Schreiner S., Hardt M., Moebus A., Santoso S., Bindereif A. (2018). Selective release of circRNAs in platelet-derived extracellular vesicles. J. Extracell. Vesicles.

[103] Chabert A., Hamzeh-Cognasse H., Pozzetto B., Cognasse F., Schattner M., Gomez R.M., Garraud O. (2015). Human platelets and their capacity of binding viruses: Meaning and challenges?. BMC Immunol..

[104] Hottz E.D., Bozza F.A., Bozza P.T. (2018). Platelets in Immune Response to Virus and Immunopathology of Viral Infections. Front. Med. (Lausanne).

[105] Real F., Capron C., Sennepin A., Arrigucci R., Zhu A., Sannier G., Zheng J., Xu L., Masse J.M., Greffe S. (2020). Platelets from HIV-infected individuals on antiretroviral drug therapy with poor CD4(+) T cell recovery can harbor replication-competent HIV despite viral suppression. Sci. Transl. Med..

[106] Simpson S.R., Singh M.V., Dewhurst S., Schifitto G., Maggirwar S.B. (2020). Platelets function as an acute viral reservoir during HIV-1 infection by harboring virus and T-cell complex formation. Blood Adv..

[107] Simon A.Y., Sutherland M.R., Pryzdial E.L. (2015). Dengue virus binding and replication by platelets. Blood.

[108] Tang N., Li D., Wang X., Sun Z. (2020). Abnormal coagulation parameters are associated with poor prognosis in patients with novel coronavirus pneumonia. J. Thromb. Haemost..

[109] Zaid Y., Puhm F., Allaeys I., Naya A., Oudghiri M., Khalki L., Limami Y., Zaid N., Sadki K., Ben El Haj R. (2020). Platelets can contain SARS-CoV-2 RNA and are hyperactivated in COVID-19. medRxiv.

[110] Manne B.K., Denorme F., Middleton E.A., Portier I., Rowley J.W., Stubben C.J., Petrey A.C., Tolley N.D., Guo L., Cody M.J. (2020). Platelet Gene Expression and Function in COVID-19 Patients. Blood.

[111] Fisher M.H., Di Paola J. (2018). Genomics and transcriptomics of megakaryocytes and platelets: Implications for health and disease. Res. Pract. Thromb. Haemost..

[112] Weyrich A.S., Lindemann S., Tolley N.D., Kraiss L.W., Dixon D.A., Mahoney T.M., Prescott S.P., McIntyre T.M., Zimmerman G.A. (2004). Change in protein phenotype without a nucleus: Translational control in platelets. Semin. Thromb. Hemost..

[113] Weyrich A.S., Zimmerman G.A. (2003). Evaluating the relevance of the platelet transcriptome. Blood.

[114] Lindemann S., Tolley N.D., Eyre J.R., Kraiss L.W., Mahoney T.M., Weyrich A.S. (2001). Integrins regulate the intracellular distribution of eukaryotic initiation factor 4E in platelets. A checkpoint for translational control. J. Biol. Chem..

[115] Lindemann S., Tolley N.D., Dixon D.A., McIntyre T.M., Prescott S.M., Zimmerman G.A., Weyrich A.S. (2001). Activated platelets mediate inflammatory signaling by regulated interleukin 1beta synthesis. J. Cell Biol..

[116] Shim M.H., Hoover A., Blake N., Drachman J.G., Reems J.A. (2004). Gene expression profile of primary human CD34+CD38lo cells differentiating along the megakaryocyte lineage. Exp. Hematol..

[117] Kim H.L. (2003). Comparison of oligonucleotide-microarray and serial analysis of gene expression (SAGE) in transcript profiling analysis of megakaryocytes derived from CD34+ cells. Exp. Mol. Med..

[118] Cecchetti L., Tolley N.D., Michetti N., Bury L., Weyrich A.S., Gresele P. (2011). Megakaryocytes differentially sort mRNAs for matrix metalloproteinases and their inhibitors into platelets: A mechanism for regulating synthetic events. Blood.

[119] Flaumenhaft R. (2011). Platelets get the message. Blood.

[120] Nilsson R.J., Karachaliou N., Berenguer J., Gimenez-Capitan A., Schellen P., Teixido C., Tannous J., Kuiper J.L., Drees E., Grabowska M. (2016). Rearranged EML4-ALK fusion transcripts sequester in circulating blood platelets and enable blood-based crizotinib response monitoring in non-small-cell lung cancer. Oncotarget.

[121] Cashell A.W., Buss D.H. (1992). The frequency and significance of megakaryocytic emperipolesis in myeloproliferative and reactive states. Ann. Hematol..

[122] McGinnis E., Chipperfield K.M. (2019). Striking emperipolesis in megakaryocytes of gray platelet syndrome. Blood.

[123] Cunin P., Bouslama R., Machlus K.R., Martinez-Bonet M., Lee P.Y., Wactor A., Nelson-Maney N., Morris A., Guo L., Weyrich A. (2019). Megakaryocyte emperipolesis mediates membrane transfer from intracytoplasmic neutrophils to platelets. Elife.

[124] Georgantas R.W., Hildreth R., Morisot S., Alder J., Liu C.G., Heimfeld S., Calin G.A., Croce C.M., Civin C.I. (2007). CD34+ hematopoietic stem-progenitor cell microRNA expression and function: A circuit diagram of differentiation control. Proc. Natl. Acad. Sci. USA.

[125] Emambokus N., Vegiopoulos A., Harman B., Jenkinson E., Anderson G., Frampton J. (2003). Progression through key stages of haemopoiesis is dependent on distinct threshold levels of c-Myb. EMBO J..

[126] Lu J., Guo S., Ebert B.L., Zhang H., Peng X., Bosco J., Pretz J., Schlanger R., Wang J.Y., Mak R.H. (2008). MicroRNA-mediated control of cell fate in megakaryocyte-erythrocyte progenitors. Dev. Cell.

[127] Havelange V., Garzon R. (2010). MicroRNAs: Emerging key regulators of hematopoiesis. Am. J. Hematol..

[128] Weiss C.N., Ito K. (2019). microRNA-22 promotes megakaryocyte differentiation through repression of its target, GFI1. Blood Adv..

[129] Emmrich S., Henke K., Hegermann J., Ochs M., Reinhardt D., Klusmann J.H. (2012). miRNAs can increase the efficiency of ex vivo platelet generation. Ann. Hematol..

[130] Rowley J.W., Chappaz S., Corduan A., Chong M.M., Campbell R., Khoury A., Manne B.K., Wurtzel J.G., Michael J.V., Goldfinger L.E. (2016). Dicer1-mediated miRNA processing shapes the mRNA profile and function of murine platelets. Blood.

[131] Nagalla S., Shaw C., Kong X., Kondkar A.A., Edelstein L.C., Ma L., Chen J., McKnight G.S., López J.A., Yang L. (2011). Platelet microRNA-mRNA coexpression profiles correlate with platelet reactivity. Blood.

[132] Kondkar A., Bray M., Leal S., Nagalla S., Liu D., Jin Y., Dong J., Ren Q., Whiteheart S., Shaw C. (2010). VAMP8/endobrevin is overexpressed in hyperreactive human platelets: Suggested role for platelet microRNA. J. Thromb. Haemost..

[133] Tran J.Q.D., Pedersen O.H., Larsen M.L., Grove E.L., Kristensen S.D., Hvas A.M., Nissen P.H. (2020). Platelet microRNA expression and association with platelet maturity and function in patients with essential thrombocythemia. Platelets.

[134] Shi R., Zhou X., Ji W.J., Zhang Y.Y., Ma Y.Q., Zhang J.Q., Li Y.M. (2015). The Emerging Role of miR-223 in Platelet Reactivity: Implications in Antiplatelet Therapy. Biomed. Res. Int..

[135] Leierseder S., Petzold T., Zhang L., Loyer X., Massberg S., Engelhardt S. (2013). MiR-223 is dispensable for platelet production and function in mice. Thromb. Haemost..

[136] Sunderland N., Skroblin P., Barwari T., Huntley R.P., Lu R., Joshi A., Lovering R.C., Mayr M. (2017). MicroRNA Biomarkers and Platelet Reactivity: The Clot Thickens. Circ. Res..

[137] Perry R.B., Ulitsky I. (2016). The functions of long noncoding RNAs in development and stem cells. Development.

[138] Tran N.T., Su H., Khodadadi-Jamayran A., Lin S., Zhang L., Zhou D., Pawlik K.M., Townes T.M., Chen Y., Mulloy J.C. (2016). The AS-RBM15 lncRNA enhances RBM15 protein translation during megakaryocyte differentiation. EMBO Rep..

[139] Zhang L., Tran N.T., Su H., Wang R., Lu Y., Tang H., Aoyagi S., Guo A., Khodadadi-Jamayran A., Zhou D. (2015). Cross-talk between PRMT1-mediated methylation and ubiquitylation on RBM15 controls RNA splicing. Elife.

[140] Paralkar V.R., Mishra T., Luan J., Yao Y., Kossenkov A.V., Anderson S.M., Dunagin M., Pimkin M., Gore M., Sun D. (2014). Lineage and species-specific long noncoding RNAs during erythro-megakaryocytic development. Blood.

[141] Pimkin M., Kossenkov A.V., Mishra T., Morrissey C.S., Wu W., Keller C.A., Blobel G.A., Lee D., Beer M.A., Hardison R.C. (2014). Divergent functions of hematopoietic transcription factors in lineage priming and differentiation during erythro-megakaryopoiesis. Genome Res..

[142] Suslova T.E., Sitozhevskii A.V., Ogurkova O.N., Kravchenko E.S., Kologrivova I.V., Anfinogenova Y., Karpov R.S. (2014). Platelet hemostasis in patients with metabolic syndrome and type 2 diabetes mellitus: cGMP- and NO-dependent mechanisms in the insulin-mediated platelet aggregation. Front. Physiol..

[143] Samos M., Fedor M., Kovar F., Mokan M., Bolek T., Galajda P., Kubisz P., Mokan M. (2016). Type 2 Diabetes and ADP Receptor Blocker Therapy. J. Diabetes Res..

[144] Jung J.H., Tantry U.S., Gurbel P.A., Jeong Y.H. (2015). Current antiplatelet treatment strategy in patients with diabetes mellitus. Diabetes Metab. J..

[145] Zhang Y., Zhang S., Ding Z. (2017). Role of P2Y12 Receptor in Thrombosis. Adv. Exp. Med. Biol..

[146] Zhou M., Gao M., Luo Y., Gui R., Ji H. (2019). Long non-coding RNA metallothionein 1 pseudogene 3 promotes p2y12 expression by sponging miR-126 to activate platelet in diabetic animal model. Platelets.

[147] Kieffer N., Guichard J., Farcet J.P., Vainchenker W., Breton-Gorius J. (1987). Biosynthesis of major platelet proteins in human blood platelets. Eur. J. Biochem..

[148] Rosenwald I.B., Pechet L., Han A., Lu L., Pihan G., Woda B., Chen J.J., Szymanski I. (2001). Expression of translation initiation factors elF-4E and elF-2alpha and a potential physiologic role of continuous protein synthesis in human platelets. Thromb. Haemost..

[149] Weyrich A.S., Dixon D.A., Pabla R., Elstad M.R., McIntyre T.M., Prescott S.M., Zimmerman G.A. (1998). Signal-dependent translation of a regulatory protein, Bcl-3, in activated human platelets. Proc. Natl. Acad. Sci. USA.

[150] Aslan J.E., Tormoen G.W., Loren C.P., Pang J., McCarty O.J. (2011). S6K1 and mTOR regulate Rac1-driven platelet activation and aggregation. Blood.

[151] Jiang P., Lan Y., Luo J., Ren Y.L., Liu D.G., Pang J.X., Liu J., Li J., Wang C., Cai J.P. (2014). Rapamycin promoted thrombosis and platelet adhesion to endothelial cells by inducing membrane remodeling. BMC Cell Biol..

[152] López E., Berna-Erro A., Bermejo N., Brull J.M., Martinez R., Garcia Pino G., Alvarado R., Salido G.M., Rosado J.A., Cubero J.J. (2013). Long-term mTOR inhibitors administration evokes altered calcium homeostasis and platelet dysfunction in kidney transplant patients. J. Cell Mol. Med..

[153] Zimmerman G.A., Weyrich A.S. (2008). Signal-dependent protein synthesis by activated platelets: New pathways to altered phenotype and function. Arterioscler. Thromb. Vasc. Biol..

[154] Pabla R., Weyrich A.S., Dixon D.A., Bray P.F., McIntyre T.M., Prescott S.M., Zimmerman G.A. (1999). Integrin-dependent control of translation: Engagement of integrin alphaIIbbeta3 regulates synthesis of proteins in activated human platelets. J. Cell Biol..

[155] Weyrich A.S., Denis M.M., Schwertz H., Tolley N.D., Foulks J., Spencer E., Kraiss L.W., Albertine K.H., McIntyre T.M., Zimmerman G.A. (2007). mTOR-dependent synthesis of Bcl-3 controls the retraction of fibrin clots by activated human platelets. Blood.

[156] Lemaitre D., Vericel E., Polette A., Lagarde M. (1997). Effects of fatty acids on human platelet glutathione peroxidase: Possible role of oxidative stress. Biochem. Pharmacol..

[157] Gerstberger S., Hafner M., Tuschl T. (2014). A census of human RNA-binding proteins. Nat. Rev. Genet..

[158] Hentze M.W., Castello A., Schwarzl T., Preiss T. (2018). A brave new world of RNA-binding proteins. Nat. Rev. Mol. Cell Biol..

[159] Hansen M., Zeddies S., Meinders M., di Summa F., van Alphen F.P.J., Hoogendijk A.J., Moore K.S., Halbach M., Gutiérrez L., van den Biggelaar M. (2020). The RNA-Binding Protein ATXN2 is Expressed during Megakaryopoiesis and May Control Timing of Gene Expression. Int. J. Mol. Sci..

[160] Ostrowski L.A., Hall A.C., Mekhail K. (2017). Ataxin-2: From RNA Control to Human Health and Disease. Genes (Basel).

[161] Yokoshi M., Li Q., Yamamoto M., Okada H., Suzuki Y., Kawahara Y. (2014). Direct binding of Ataxin-2 to distinct elements in 3’ UTRs promotes mRNA stability and protein expression. Mol. Cell.

[162] Jin S., Mi Y., Song J., Zhang P., Liu Y. (2018). PRMT1-RBM15 axis regulates megakaryocytic differentiation of human umbilical cord blood CD34(+) cells. Exp. Ther. Med..

[163] Stoskus M., Vaitkeviciene G., Eidukaite A., Griskevicius L. (2016). ETV6/RUNX1 transcript is a target of RNA-binding protein IGF2BP1 in t(12;21)(p13;q22)-positive acute lymphoblastic leukemia. Blood Cells Mol. Dis..

[164] Elagib K.E., Lu C.H., Mosoyan G., Khalil S., Zasadzinska E., Foltz D.R., Balogh P., Gru A.A., Fuchs D.A., Rimsza L.M. (2017). Neonatal expression of RNA-binding protein IGF2BP3 regulates the human fetal-adult megakaryocyte transition. J. Clin. Investig..

[165] Stolla M.C., Catherman S.C., Kingsley P.D., Rowe R.G., Koniski A.D., Fegan K., Vit L., McGrath K.E., Daley G.Q., Palis J. (2019). Lin28b regulates age-dependent differences in murine platelet function. Blood Adv..

[166] Leon C., Eckly A., Hechler B., Aleil B., Freund M., Ravanat C., Jourdain M., Nonne C., Weber J., Tiedt R. (2007). Megakaryocyte-restricted MYH9 inactivation dramatically affects hemostasis while preserving platelet aggregation and secretion. Blood.

[167] Naarmann-de Vries I.S., Brendle A., Bahr-Ivacevic T., Benes V., Ostareck D.H., Ostareck-Lederer A. (2016). Translational control mediated by hnRNP K links NMHC IIA to erythroid enucleation. J. Cell Sci..

[168] Bramham C.R., Wells D.G. (2007). Dendritic mRNA: Transport, translation and function. Nat. Rev. Neurosci..

[169] Thelen M.P., Kye M.J. (2019). The Role of RNA Binding Proteins for Local mRNA Translation: Implications in Neurological Disorders. Front. Mol. Biosci..

[170] Wang I., Hennig J., Jagtap P.K., Sonntag M., Valcarcel J., Sattler M. (2014). Structure, dynamics and RNA binding of the multi-domain splicing factor TIA-1. Nucleic Acids Res..

[171] Corduan A., Ple H., Laffont B., Wallon T., Plante I., Landry P., Provost P. (2015). Dissociation of SERPINE1 mRNA from the translational repressor proteins Ago2 and TIA-1 upon platelet activation. Thromb. Haemost..

[172] Schwertz H., Rowley J.W., Schumann G.G., Thorack U., Campbell R.A., Manne B.K., Zimmerman G.A., Weyrich A.S., Rondina M.T. (2018). Endogenous LINE-1 (Long Interspersed Nuclear Element-1) Reverse Transcriptase Activity in Platelets Controls Translational Events Through RNA-DNA Hybrids. Arterioscler. Thromb. Vasc. Biol..

[173] Auerbach E., Aboulafia D.M. (2012). Venous and arsterial thromboembolic complications associated with HIV infection and highly active antiretroviral therapy. Semin. Thromb. Hemost..

